# Migration of Hepatocellular Carcinoma Into the Right Atrium

**DOI:** 10.1016/j.jaccas.2024.102857

**Published:** 2024-12-18

**Authors:** Emaad Siddiqui, Pallavi Solanki, Christine Gerula, Marc Klapholz, Alfonso H. Waller

**Affiliations:** Division of Cardiology, Department of Medicine, Rutgers New Jersey Medical School, Newark, New Jersey, USA

**Keywords:** cancer, computed tomography, echocardiography, inferior vena cava, metastases, right atrium

## Abstract

A 40-year-old man with a medical history of hepatitis B presented with abdominal distention and leg swelling. A computed tomography scan of the abdomen revealed cirrhosis and a large mass extending from the liver into the inferior vena cava and extending into the right atrium. A transthoracic echocardiogram revealed a large right atrial mass extending from the inferior vena cava with possible attachment to the interatrial septum. The patient was eventually determined to have advanced hepatocellular carcinoma with invasion into the inferior vena cava and into the right atrium, along with pulmonary metastases.

## Graphical Abstract

**Figure:**
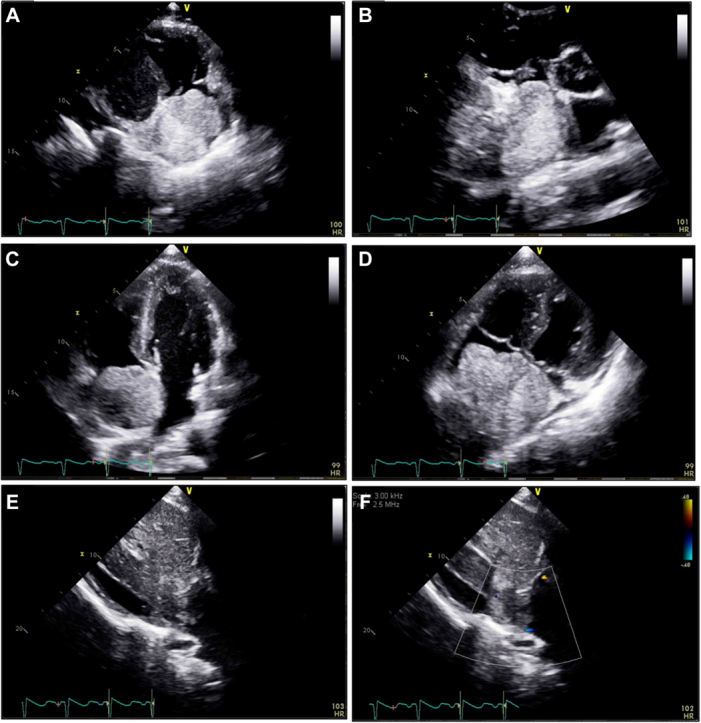

A 40-year-old man with a medical history of hepatitis B presented with abdominal distention and leg swelling. On admission, he was found to be tachycardic with abdominal distention and lower extremity edema. An ultrasound scan of the abdomen revealed cirrhosis and a large mass extending from the liver into the inferior vena cava (IVC) and extending into the right atrium (RA). A transthoracic echocardiogram (TTE) demonstrated normal left and right ventricular systolic function and no pericardial effusion. The TTE also revealed a 6.4 cm × 4.8 cm echogenic mass in the (RA (Figures 1A and 1B). The mass nearly obliterated the RA, with shifting of the interatrial septum and possible attachment (Figures 1C and 1D). The mass appeared to arise from the proximal portion of the IVC (Figure 1E), and color Doppler, in the views obtained, showed minimal flow from the IVC to the RA (Figure 1F). There was no evidence of direct tricuspid valve involvement or pseudo-tricuspid valve stenosis.

**Take-Home Messages**

• HCC is an aggressive cancer, and given the proximity of the liver to the IVC, extension into the RA is a rare but known complication of this tumor.
• This case highlights that imaging may identify cardiac masses arising from the IVC.

**Figure 1:**
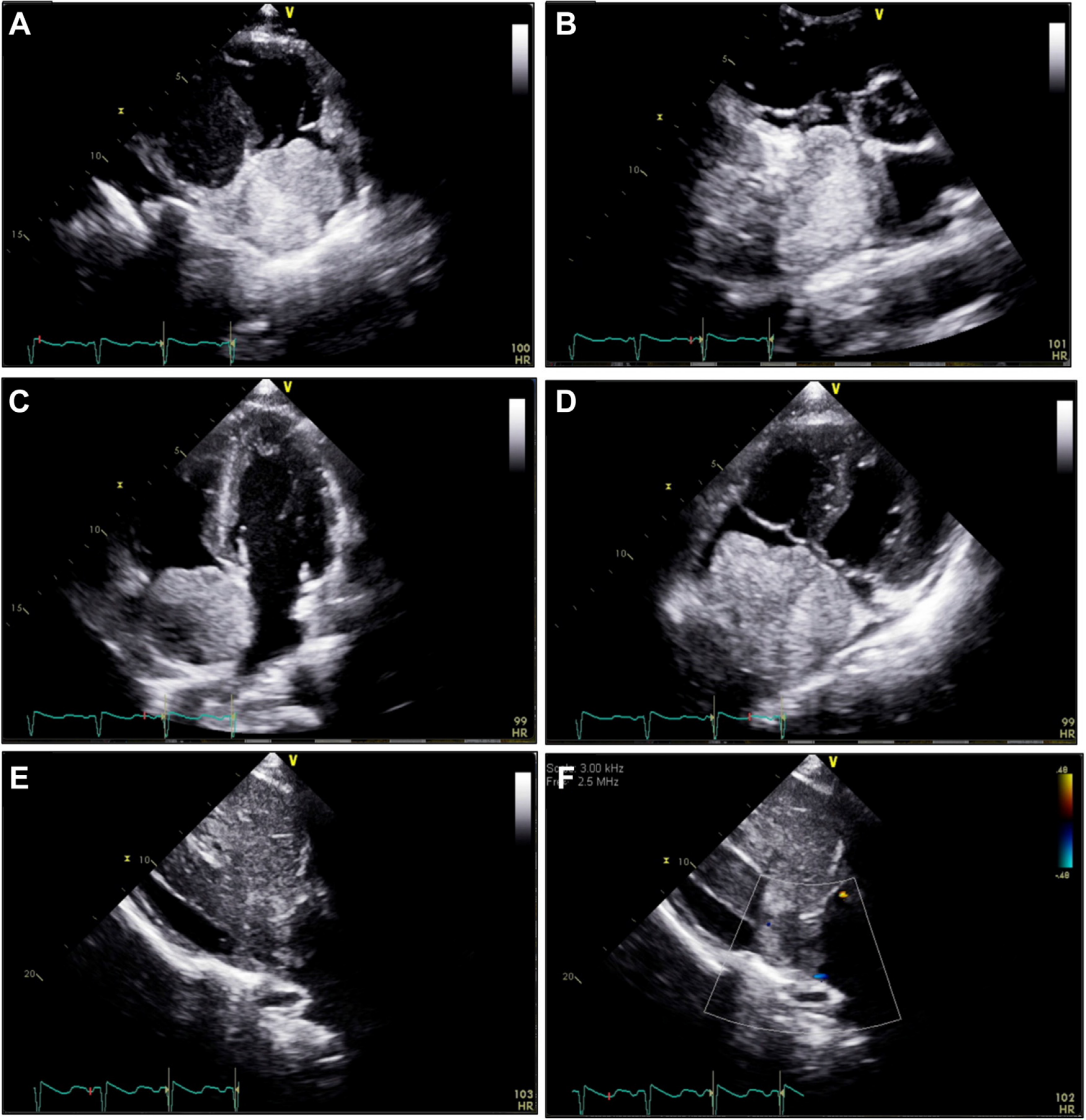
Hepatocellular Carcinoma in the Right Atrium (A) Right ventricular inflow view demonstrating the right atrial mass occupying the entire right atrium. (B) Parasternal short-axis view with a focus on the right atrium demonstrating the mass. (C) Apical 4-chamber view both on axis with the left ventricle and (D) off axis with a focus on the right ventricle demonstrating the right atrial mass with possible attachment to the interatrial septum. (E) In the subcostal view, the right atrial mass appeared to have originated from the inferior vena cava, and (F) color Doppler demonstrated minimal flow to the right atrium because of the mass, causing obstruction.

A computed tomography scan of the chest demonstrated an ill-defined, enhancing liver mass with extension into the hepatic vein, IVC, and cavoatrial junction with associated thrombi (Video 1). The imaging findings were suggestive of hepatocellular carcinoma (HCC), and blood testing demonstrated an elevated α-fetoprotein level to 143,814 ng/mL, which was consistent with the diagnosis of HCC. He was also found to have metastatic disease to the lungs. Given his extensive disease, the patient was a poor candidate for curative or palliative therapy, and he elected to transition to home hospice care. The patient died 3 weeks after his discharge from the hospital.

Tumor extension into the IVC or RA is an extremely rare but known complication of HCC, with an incidence reported from 0.67% to 4.1%.^1^ Although most cases are found incidentally, some may manifest with symptoms of right-sided heart failure, including tachycardia and lower extremity edema, secondary to the obstruction of blood flow into the RA or compression of the IVC.^1^^,^^2^ These symptoms are often missed because they may be attributed to comorbid conditions associated with HCC, including cirrhosis causing ascites and variceal bleeding.^2^ Although the diagnosis of HCC is often made using a combination of clinical history, imaging, and blood tests, diagnostic tools such as cardiac magnetic resonance (CMR) imaging could be helpful to characterize cardiac masses and assess for cardiac invasion. CMR was not used for this patient because he elected hospice care.

Although most patients who present with intracardiac extension of HCC have an overall poor prognosis, they generally do not die of complications of the cardiac mass itself and more often die as a result of the overall tumor burden or of liver failure.^3^ However, in our patient, the overall size of the mass may have eventually led to obstructive shock and pseudo-tricuspid stenosis. In patients with isolated HCC extending into the RA, if surgery is considered, it typically is a complex procedure that is usually associated with a poor prognosis.^3^ Additionally, surgery for an intracardiac mass is not curative if there is metastatic disease elsewhere. Therapy for this condition is often limited to less invasive therapies such as local radiotherapy, transcatheter arterial chemoembolization, and immunotherapy.^2^^,^^3^ Given the rarity of this presentation, little is known of surgical and medical outcomes, and this case therefore highlights the importance of further research into this group of patients.^2^

## Funding Support and Author Disclosures

The authors have reported that they have no relationships relevant to the contents of this paper to disclose.

## Appendix

Video 1 Computed Tomography of the Abdomen: Liver Mass Extending Into the Right Atrium. Noncontrast chest computed tomography reveals a solid tissue mass in the right lower lobe, concurrent with a hypodense lesion in the left atrium. Coronal sections on computed tomography of the abdomen demonstrating cirrhosis and a large, ill-defined hepatic mass that encases and invades the inferior vena cava, with extension into the right atrium. The mass was also associated with extensive thrombi in the portal vessels, hepatic vein, and intrahepatic inferior vena cava
